# Characterizing the *Pyrenophora teres* f. *maculata*–Barley Interaction Using Pathogen Genetics

**DOI:** 10.1534/g3.117.043265

**Published:** 2017-06-28

**Authors:** Steven A. Carlsen, Anjan Neupane, Nathan A. Wyatt, Jonathan K. Richards, Justin D. Faris, Steven S. Xu, Robert S. Brueggeman, Timothy L. Friesen

**Affiliations:** * Department of Plant Pathology, North Dakota State University, Fargo, North Dakota 58102; † Genomics and Bioinformatics Program, Department of Plant Science, North Dakota State University, Fargo, North Dakota 58102; ‡ United States Department of Agriculture-Agricultural Research Service, Cereal Crops Research Unit, Northern Crop Science Laboratory, Fargo, North Dakota 58102

**Keywords:** *Pyrenophora teres*, barley, fungal pathogen, spot form net blotch, virulence

## Abstract

*Pyrenophora teres* f. *maculata* is the cause of the foliar disease spot form net blotch (SFNB) on barley. To evaluate pathogen genetics underlying the *P. teres* f. *maculata*–barley interaction, we developed a 105-progeny population by crossing two globally diverse isolates, one from North Dakota and the other from Western Australia. Progeny were phenotyped on a set of four barley genotypes showing a differential reaction to the parental isolates, then genotyped using a restriction site-associated-genotype-by-sequencing (RAD-GBS) approach. Genetic maps were developed for use in quantitative trait locus (QTL) analysis to identify virulence-associated QTL. Six QTL were identified on five different linkage groups and individually accounted for 20–37% of the disease variation, with the number of significant QTL ranging from two to four for the barley genotypes evaluated. The data presented demonstrate the complexity of virulence involved in the *P. teres* f. *maculata*–barley pathosystem and begins to lay the foundation for understanding this important interaction.

Barley net blotch has two distinct forms, the net form net blotch (NFNB) caused by *Pyrenophora teres* f. *teres* and spot form net blotch (SFNB) caused by *P. teres* f. *maculata* (Smedegård-Petersen 1971). *P. teres* f. *maculata* can be distinguished from *P*. *teres* f. *teres* due to its characteristic spot-like necrotic lesions exhibited on the leaves of infected susceptible barley plants (Smedegård-Petersen 1971). SFNB is an increasingly important foliar disease, as the distribution and impact of SFNB has expanded in major barley-producing regions including the United States, Australia, Canada, Denmark, South Africa, and Norway (Liu *et al.* 2011; Lartey *et al.* 2013). SFNB results in many quality-reducing traits including reduced kernel size, bulk density, and plumpness, all of which contribute negatively to malt and feed quality (Liu *et al.* 2011; Wang *et al.* 2015). SFNB outbreaks typically produce yield losses of 10–40%, with complete yield losses possible when susceptible varieties are planted under environmental conditions conducive to disease (Shipton *et al.* 1973; McLean *et al.* 2009; Liu *et al.* 2011). Recent work examining yield losses due to SFNB under field conditions in North Dakota showed a 0.77% decrease in yield for every 1% increase in disease even under low disease pressure (Kinzer 2015). The findings at high disease pressure were very similar in studies from Australia (Jayasena *et al.* 2007).

Both forms of *P. teres* demonstrate a high level of virulence diversity, making disease resistance breeding difficult [reviewed in Liu *et al.* (2011)]. Several prior studies that include *P*. *teres* f. *maculata* collections illustrate the diversity of virulence observed in *P*. *teres* f. *maculata* globally (Karki and Sharp 1986; Tekauz 1990; Arabi *et al.* 1992; Gupta *et al.* 2011; McLean *et al.* 2012). Karki and Sharp (1986) used 20 barley cultivars to evaluate 14 *P*. *teres* f. *maculata* isolates from regions of the Mediterranean (Morocco, Tunisia, and Turkey) and Montana. Shared and differential host responses were observed among the lines inoculated with isolates from the two regions. Tekauz (1990) evaluated a collection of *P*. *teres* f. *maculata* isolates on 12 barley differentials, three of which were in common with the Karki and Sharp (1986) study. Tekauz (1990) described 20 pathotypes of *P*. *teres* f. *maculata*, illustrating the diversity represented in the Canadian *P*. *teres* f. *maculata* population. More recently, McLean *et al.* (2012) evaluated 19 *P*. *teres* f. *maculata* isolates collected from Canada and Australia on a diverse set of 95 barley lines. The lines evaluated at the seedling stage exhibited a variety of disease responses, with 91 of the lines exhibiting a differential response between the Canadian and Australian isolates. The variety of disease responses demonstrated in this study express the complex level of pathogen virulence present in the worldwide pathogen population. Even with the number of virulences/avirulences identified globally there is still little known about the genes and potential necrotrophic effectors (NEs) involved in the pathosystem.

Through the use of biparental host mapping populations, QTL associated with SFNB resistance/susceptibility have been reported on all barley chromosomes, with major QTL identified on barley chromosmomes 2H (Williams *et al.* 1999), 4H (Friesen *et al.* 2006; Grewal *et al.* 2008), 5H (Manninen *et al.* 2006), 6H (Grewal *et al.* 2008), and 7H (Williams *et al.* 1999, 2003; Grewal *et al.* 2008). Williams *et al.* (1999) reported *Rpt4* as a single dominant resistance gene that mapped to barley chromosome 7H. *Rpt4* was determined to account for between 27 and 74% of the phenotypic variation in the Galleon × Haruna Nijo DH population. Molnar *et al.* (2000) reported two genomic regions on chromosome 2H associated with SFNB resistance in the doubled haploid population Leger × CI 9831. Friesen *et al.* (2006) reported a QTL on barley chromosome 4H accounting for 64% of the disease variation in the doubled haploid population SM89010 × Q21861. Grewal *et al.* (2008) identified three QTL using the doubled haploid population CDC Dolly × TR251, which were designated *QRpts4*, *QRpt6*, and *QRpt7*, and mapped to barley chromosomes 4H, 6H, and 7H, respectively, with effects ranging from 8 to 21% of disease variation. Using the doubled haploid population Rolfi × CI 9819, *Rpt6* was mapped to barley chromosome 5H and explained 65–84% of the disease variation (Manninen *et al.* 2006).

More recently, genome-wide association studies (GWAS) have been used to identify genomic regions associated with resistance/susceptibility. Tamang *et al.* (2015) used SFNB disease reaction data (Neupane *et al.* 2015) and genotypic data (Muñoz-Amatriaín *et al.* 2014) from a 1480-line barley core collection to identify 27 distinct loci associated with SFNB resistance/susceptibility. Among the 27 loci identified in GWAS, six loci were consistent with previously identified QTL (Tamang *et al.* 2015). Similarly, Wang *et al.* (2015) identified 29 marker trait associations (MTA) that represented 24 unique regions among 898 breeding lines from the Northern Region Barley Breeding Program in Australia. Five of the MTA regions identified from this AM study were similar to QTL identified in previous biparental mapping studies. Both GWAS studies illustrate the numerous genomic regions that are involved in SFNB resistance/susceptibility. Given the number of QTL and MTA identified in the literature, it is likely that the corresponding virulence/avirulence in the pathogen is similarly complex.

*P. teres* is a heterothalic ascomycete in the Dothideomycete class. The heterothallic nature of this pathogen and the ability to make crosses in the laboratory make this pathogen a good model for studying the genetics of virulence. Three previous studies have used *P. teres* f. *teres* crosses to identify qualitative interactions associated with single barley lines (Beattie *et al.* 2007; Weiland *et al.* 1999; Lai *et al.* 2007). A fourth study used a *P. teres* f. *teres* cross to show that virulence can also be inherited in a quantitative manner (Shjerve *et al.* 2014), similar to what has been shown on the host side (Liu *et al.* 2015). However, no similar studies have been published characterizing the virulence of *P. teres* f. *maculata*.

As seen above, the majority of our current understanding of the *P*. *teres* f. *maculata*–barley interactions comes from the host side, where researchers have identified genomic regions involved in SFNB resistance/susceptibility. To begin filling the knowledge gaps on the genetics of pathogen virulence, a biparental mapping population of the pathogen was developed using a cross of a North American isolate with an Australian isolate of *P*. *teres* f. *maculata*. This population was used to genetically characterize and localize genomic regions associated with *P. teres* f. *maculata* virulence/avirulence.

## Materials and Methods

### Biological materials

*Pyrenophora teres* f. *maculata* isolate FGOB10Ptm-1 (FGO) was collected from North Dakota, and isolate SG1 was collected from Western Australia and provided by Simon Ellwood, Curtin University. A biparental mapping population consisting of 105 progeny was generated from a cross of FGO and SG1 in a manner similar to Shjerve *et al.* (2014). Strains are available upon request. Briefly, sterilized wheat straw was placed on Sach’s Agar media plates (1 g CaNO_3_, 0.25 g MgSO_4_ 7H_2_O, trace FeCl_3_, 0.25 g K_2_HPO, 4 g CaCO_3_, 20 g agar, and ddH_2_O to 1 L). Parental isolates were grown on V8 Potato Dextrose Agar media plates (150 ml V8 juice, 10 g Difco PDA, 3 g CaCO_3_, 10 g agar, and ddH_2_O to 1 L) in the dark for 7 d at room temperature, followed by 24 hr light at room temperature then 24 hr dark at 13°. Plates that contained conidia were flooded with water and scraped with an inoculating loop to obtain a spore suspension that was diluted to 4000 spores/ml. A 100 µl aliquot of the prepared spore suspension from FGO and SG1 was pipetted onto opposite ends of the stems on the Sach’s media plates. By gently rocking the plates, the suspensions converged at the centers of the stems. Plates were incubated in the dark at 15° for 12 d and then moved to 13° under a 12 hr photoperiod until pseudothecia matured and asci with ascospores were observed (∼12 wk), at which time single stems were transferred to the lids of petri dishes containing water agar and placed upside down under the same light and temperature conditions, and checked regularly for ejected ascospores. Water agar plates were observed for single asci discharges (eight ascospores) and collected as single ascospores to minimize the potential for collecting clones resulting from the mitotic division from a single ascus. Isolated ascospores were allowed to mature into conidia-producing colonies. A small portion of the colony was collected and spread across water agar to allow for the isolation of single conidia. Individual conidia were placed on V8-PDA media plates to produce sporulating colonies followed by another single spore isolation to ensure the genetic purity of the progeny. Single spore progeny were grown out on V8-PDA plates, dried in 8 mm plugs, and stored at −20°.

Differential barley genotypes assessed in this study included three lines frequently used in SFNB differential sets, Skiff, 81-82/033, and TR326 (McLean *et al.* 2014), as well as the barley line PI 392501 shown by Neupane *et al.* (2015) to have a strong differential reaction between FGO (higher virulence) and SG1 (lower virulence). The parental isolate FGO is virulent on Skiff, 81-82/033, TR326, and PI 392501 whereas, SG1 has significantly reduced virulence on these four lines.

### Disease evaluation

As it was logistically impossible to inoculate all 105 progeny isolates at once, eight progeny were prepared and inoculated in a single day. Spores of the eight isolates were harvested and counted over the span of ∼1.5 hr and, once harvested, were stored at 4° until inoculation. Inoculations were completed in a span of ∼1 hr. Two sets of eight isolates were completed per week, overall taking ∼7 wk per complete replicate. Three replicates were performed for each parental and progeny isolate across the set of barley lines, with all replicates being completed in ∼4 months. Checks including Tradition (moderately susceptible), PI67381 (resistant) (Neupane *et al.* 2015), and Pinnacle (susceptible) were monitored closely to maintain as much consistency as possible. Replicates where the checks were visibly over or underinoculated were discarded and repeated. Barley lines were arranged in racks of 49 cone-tainers (Steuwe and Sons, Corvallis, OR) with the order of the lines and isolate blocks being randomized between replicates. Inoculated barley lines were surrounded by a border row of Tradition barley to reduce any edge effect. Three barley seeds were sown per cone with two cones planted per replicate. Seeds were sown into Metro Mix 902 soil (Sun Gro Horticulture). Plant material was grown under greenhouse conditions to the two-leaf stage (∼14 d) prior to inoculation but the remainder of the experiment was completed in an environmentally controlled growth chamber (Purcival, Perry, IA) in order to reduce the environmental variability of the greenhouse.

To produce spore inoculum for disease evaluations, dried mycelial plugs of the parental isolates and 105 progeny were grown, and spores were harvested by flooding plates with ∼50 ml of sterile ddH_2_O and releasing spores by scraping the plates with a sterile inoculating loop. The harvested spore suspension was collected and adjusted to 2000 spores/ml for inoculation. Tween 20 was added at 1 drop (30 µl) per 50 ml of spore suspension to reduce surface tension and allow for even inoculum dispersal on plant leaves. Plants were inoculated according to Neupane *et al.* (2015). Inoculated plants were then placed in a humidity chamber for 24 hr under constant light at 100% relative humidity. After 24 hr, plants were moved to an environmentally controlled growth chamber under a 12 hr photoperiod at 21° for 6 d. Disease levels of infected plants were evaluated 7 d postinoculation using the 1–5 rating scale described in Neupane *et al.* (2015). Virulence in this study is defined as the amount of disease resulting from a specific isolate–host line interaction.

### Fungal DNA extraction

DNA was extracted for use in a two-enzyme RAD-GBS protocol as follows. Six 8 mm diameter dried agar plugs were inoculated into a covered 250 ml Erlenmeyer flask containing 60 ml of sterile Fries media [5 g (NH_4_)_2_C_4_H_4_O_6_, 1 g NH_4_NO_3_, 0.5 g MgSO_4_ * 7H_2_O, 1.3 g KH_2_PO_4_, 5.48 g K_2_HPO_4_ * 3H_2_O, 30 g sucrose, 1 g yeast extract, 2 ml trace element stock solution (167 mg LiCl, 107 mg CuCl * H_2_O, 34 mg H_2_MoO_4_, 72 mg MnCl_2_ * 4H_2_O, 80 mg CoCl_2_ * 4H_2_O, and ddH_2_O to 1 L), and ddH_2_O to 1 L]. The flask containing plugs and media was placed in a 27° incubator with shaking at 100 rpm for 5–7 d. The contents of the flask were blended using a sterile Waring blender cup and divided equally into two new media flasks containing 60 ml of sterile Fries media and returned to the incubator for 24 hr. The tissue was harvested using Miracloth (Millipore, Billerica, MA) followed by rinsing with sterile ddH_2_O. Rinsed tissue was placed in a Büchner funnel lined with Fisher brand P5 filter paper (Thermo Fisher Scientific, Waltham, MA) and vacuum dehydrated until all excess liquid was removed. The subsequent mycelial mat was frozen at −20° and placed in a lyophilizer until completely dried (∼24 hr).

Approximately 50 mg of dried mycelial tissue of each isolate was placed in 2 ml microcentrifuge tubes and ground to a fine powder with a plastic pestle (Bel-Art, Wayne, NJ) and a bench-top homogenizer using liquid nitrogen. A 750 µl aliquot of 2 M TrisCTAB (7.3 ml 0.96 M sorbitol, 5.5 ml 2 M Tris HCl pH 8.0, 2.2 ml 0.5 M EDTA pH 8.0, 10 ml 4 M NaCl, 20 ml 2% CTAB, and 5 ml 10% sarcosine) extraction buffer was added to each tube of ground tissue along with 7 units of RNase A. Tubes were placed in a 65° water bath for 2 min followed by mixing by pipetting and vortexing until the solution was completely homogenized. The solution was incubated at 65° for 45 min followed by centrifugation at 16,300 × *g* for 8 min. Supernatant from each of the samples was transferred to new 1.7 ml tubes. Next, 600 µl of a phenol:chloroform:iso-amyl alcohol (25:24:1) solution was added and inverted until evenly mixed. The mixture was centrifuged at 16,300 × *g* for 15 min. After centrifugation, the aqueous layer was transferred to new 1.7 ml tubes and mixed with 0.1 vol of 3 M NaOAc (pH 5.2) and an equal volume of isopropanol. The solution was inverted 50 times and centrifuged for 10 min at 16,300 × *g*. The pellet was rinsed with 500 µl of 70% ethanol and centrifuged at 6000 × *g* for 10 min. Residual ethanol was removed from the pellet and tubes were placed in a laminar flow hood until dry. Dried pellets of each progeny isolate were resuspended in 60 µl of molecular biology grade water.

### SNP development and RAD-GBS

RAD-GBS libraries were created as described in Leboldus *et al.* (2014). Briefly, 600 ng of gDNA per progeny isolate was digested using 16 units of *Hha*I enzyme (NEB, Ipswich, MA) for 3.5 hr at 37°. Samples were then digested with 7.5 units of *Ape*KI enzyme (NEB) with incubation at 70° for 3.5 hr. A 100 µl aliquot of molecular biology grade water was added to each digestion reaction, raising the total reaction volume to 200 µl. Reactions were extracted with an equal volume of phenol:chloroform:isoamyl alcohol (24:24:1). The aqueous phase was placed into a new 1.7 ml tube and precipitated with 3 M NaOAc and 2.5 vol of 95% ethanol. Samples were incubated at −20° for 1 hr then centrifuged at 16,300 × *g* for 20 min at 4°. Pelleted gDNA was washed with 70% ethanol and centrifuged at 16,300 × *g* for 20 min at 4°. Tubes containing gDNA pellets were placed in a laminar flow hood for drying until pellets turned clear (∼15 min). The gDNA was resuspended with 1.5 units of T4 DNA ligase (NEB) in a solution with final concentrations of 1× DNA ligase buffer, 100 µM *P*1-*Hha*I adaptors, and 100 µM of an *Ape*KI unique barcoded adaptor. Barcoded progeny were pooled into one tube containing 40 progeny libraries. These pooled libraries were column purified to filter unligated adapters. Filtered libraries were loaded onto a Pippin Prep size selector (Sage Science, Beverly, MA) and 275 bp fragments were selected for use in a 2% precast gel cassette. A 2 µl aliquot of the size-selected DNA was amplified for use as a template for the sequencing library. Amplification was conducted on a Dyad thermocycler (Bio-Rad, Hercules, CA) with the following parameters: 95° for 1 min; eight cycles of 95° for 30 sec, 62° for 30 sec, and 72° for 30 sec; followed by 72° for 5 min. The amplified library was then diluted to 2 pg/µl and used in an emulsion PCR reaction to attach monoclonal DNA template to individual Ion Sphere Particles (ISP) using the Ion Torrent One Touch 2 System (Life Technologies, Carlsbad, CA). DNA-templated ISPs were loaded onto a 318 Ion Torrent microprocessing chip and sequenced using the Ion Torrent PGM. Barcoded DNA samples from 40 progeny isolates were sequenced in parallel on each 318 chip along with both parental isolates FGO and SG1. FGO and SG1 were sequenced in each of the subsequent runs to develop a more robust reference sequence to improve the identification of SNPs in the progeny isolates. A sequencing depth of 100,000 reads per isolate was targeted to maintain marker coverage across all isolates.

### SNP identification and genetic mapping

Sequence data from Ion Torrent RAD-GBS sequencing followed a similar data workflow as described in Leboldus *et al.* (2014). Sequenced progeny isolates were sorted according to specific barcode adaptor sequences. A *de novo* assembly of FGO parental reads was conducted using the CLC Genomics Workbench (QIAGEN, Hilden, Germany) with default assembly parameters. Burrows–Wheeler Alignment Tool (BWA) (Li and Durbin 2009) utilizing the “mem” algorithm was used to align progeny sequence to the *de novo* assembly of FGO. BWA output was converted to BAM files and SNPs were identified utilizing SAMtools “mpileup” command with default settings (Li and Durbin 2009; Li 2011). The called SNPs were filtered using VCFtools requiring a single SNP call to have a genotype quality of ≥10 and be supported by three or more reads. SNP marker data were imported into Microsoft Excel and filtered to eliminate markers containing >30% missing data. Markers were further filtered based on allele frequency where markers possessing 25–75% of a single allelic state among the progeny were retained in the data set.

Postfiltered SNP marker data were exported to MapDisto 1.7.7 (Lorieux 2012) to generate a genetic linkage map of *P. teres* f. *maculata*. The FGO × SG1 genetic map was constructed in a manner similar to Shjerve *et al.* (2014). Briefly, using the SNP markers identified, linkage groups were created using the “find groups” command with a LOD threshold setting of 7.0 and an *r*-max setting of 0.3 under the Kosambi mapping function. Marker order was determined using the “check inversions” and “ripple” commands to ascertain the best marker order. Markers that mapped poorly or expanded an interval by >3 cM were removed utilizing the “drop locus command” to help produce high-quality linkage groups. *P. teres* f. *maculata* linkage groups identified in this study were compared to the close relative *P. tritici-repentis*. Using the reference genome and optical map of *P. tritici-repentis*, *P. teres* f. *maculata* linkage groups were named based on the best synteny with *P. tritici-repentis* chromosomes (Manning *et al.* 2013). An example of naming can be illustrated with two of the largest *P. teres* f. *maculata* linkage groups, where markers from these linkage groups were used as a BLASTn query to search the *P. tritici-repentis* genome for synteny. The majority of these markers had strong hits to *P. tritici-repentis* chromosome 1 and therefore these *P. teres* f. *maculata* linkage groups were named LG 1.1 and LG 1.2. SNP IDs and marker sequences are available in Supplemental Material, Table S1.

### QTL analysis

QTL analysis was conducted as described in Shjerve *et al.* (2014) with slight modifications. Briefly, average phenotypic disease values of three replicates were measured for significant associations with SNP marker loci identified using Qgene 4.3.10 (Joehanes and Nelson 2008). The initial critical LOD threshold for the FGO × SG1 fungal population was determined using 1000 permutations; permutations were performed on each of the barley genotypes with the highest resulting LOD at *P* = 0.05 being selected as the threshold for all barley genotypes. To focus on the most significant QTL, a *P* = 0.01 was also used resulting in a more stringent LOD significance threshold. Significant QTL were identified using composite interval mapping and the default settings for selection of cofactors.

### Statistical analysis

A normality test for distribution of disease reactions for each of the barley lines inoculated with the FGO × SG1 population was performed using the Shapiro–Wilk test under the PROC UNIVARIATE procedure of SAS program version 9.3 (SAS Institute, Cary, NC). Based on a normality test, the Barlett’s *χ*^2^ and Levene’s tests for normal and nonnormal distribution at *P* = 0.01 with 2 degrees of freedom was chosen to determine the homogeneity of disease reaction variances among the three replicates. Homogeneity was tested under the general linear model procedure using SAS version 9.3. Replicates found to be homogeneous were averaged and used for QTL analysis.

Markers identified using the “select cofactor” command as described above were used for defining parental genotypes at each QTL for each progeny isolate. Genotypic classes were assembled and the average disease reactions of the genotypic classes were separated by least significant difference (LSD) at α = 0.05. If the difference between genotypic classes was greater than the calculated LSD value, the classes were considered significantly different.

### Data availability

*P. teres* f. *maculata* isolates are available upon request. Table S1 contains detailed descriptions of all markers used in this study including nucleotide sequences, linkage groups, and centimorgan locations.

## Results

### Phenotypic results

**Figure 1:**
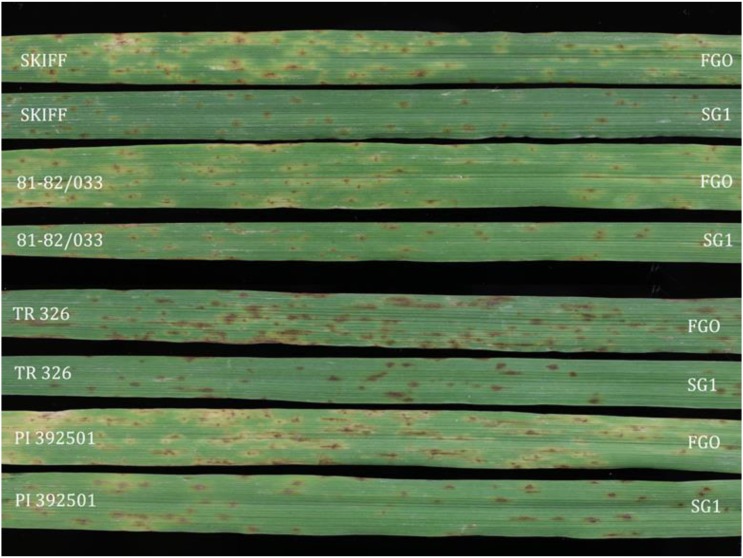
Average disease reactions of parental isolates FGO and SG1 on barley genotypes Skiff, 81-82/033, TR326, and PI 392501.

**Figure 2:**
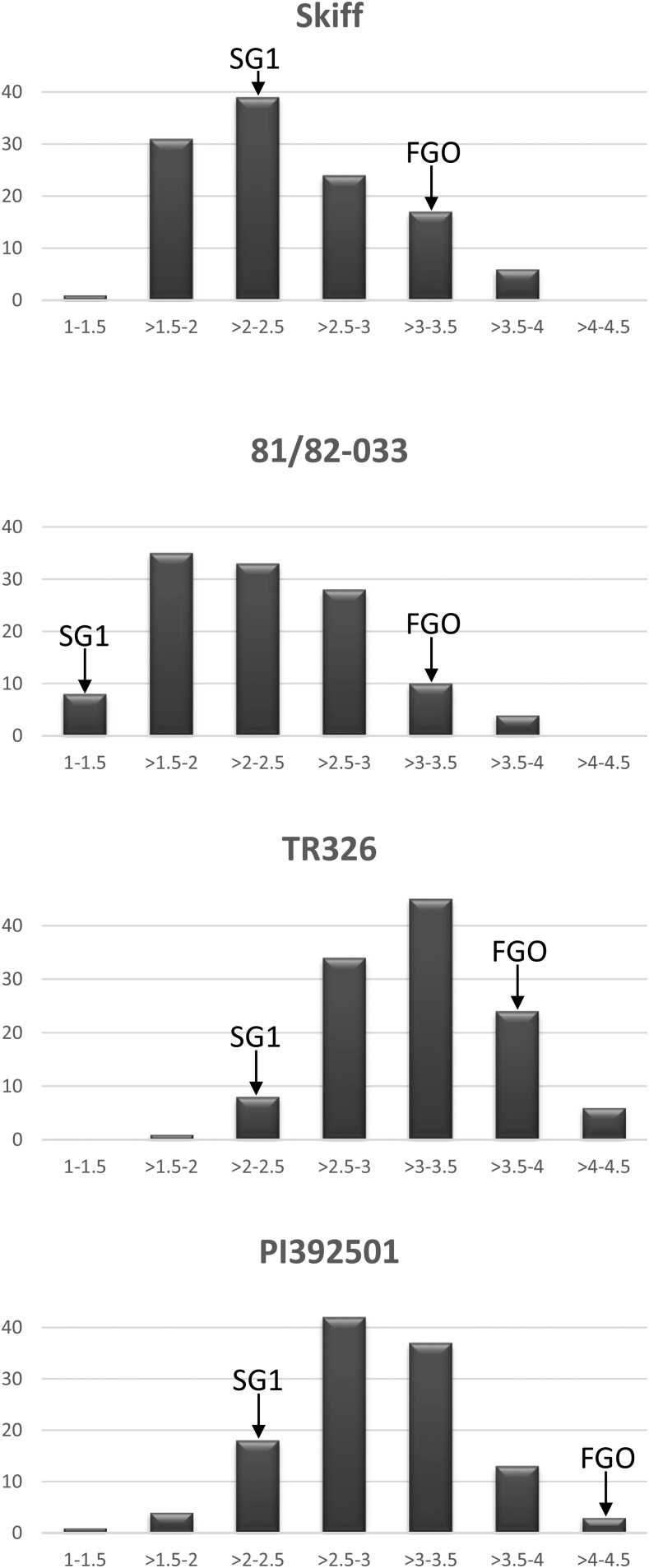
Histograms representing progeny analysis of the average disease reactions of FGO × SG1 progeny on barley genotypes Skiff, 81-82/033, TR326, and PI 392501. The *y*-axis shows the frequency of progeny, while the *x*-axis shows the average disease reaction of FGO × SG1 progeny on individual barley genotypes. Parental disease reactions are indicated on the histogram as SG1 and FGO.

Using a 1–5 SFNB disease reaction type scale (Neupane *et al.* 2015), the parental isolate SG1 caused average disease reactions of 1.5, 2.2, 2.3, and 2.5 on barley genotypes 81-82/033, Skiff, TR326, and PI 392501, respectively (Figure 1 and Table 1). The isolate FGO caused average disease reaction types of 3.2, 3.3, 3.7, and 4.2 on the same four lines, respectively, all of which were higher than SG1 (Figure 1 and Table 1). Most of the progeny showed intermediate disease reactions, but some caused average disease reactions higher than FGO or lower than SG1 indicating transgressive segregation (Figure 2).

**Table 1:** Comparison of average disease reactions of FGO and SG1 on barley genotypes

Barley Genotype	FGO	SG1
Skiff	3.3 ± 0.029	2.2 ± 0.29
81-82/033	3.2 ± 0.29	1.5 ± 0
TR326	3.7 ± 0.29	2.3 ± 0.29
PI 392501	4.2 ± 0.29	2.5 ± 0

Average disease reaction of parental *P*. *teres* f. *maculata* isolates FGO and SG1 on barley genotypes: Skiff, 81-82/033, TR326, and PI 392501. The barley genotype is shown in the first column of the table followed by the FGO average disease reaction and the SG1 average disease reaction. Average disease reactions are calculated using a 1–5 disease reaction scale with 1 being avirulent and 5 being highly virulent.

Average disease reactions of progeny isolates inoculated on Skiff ranged from 1.5 to 4.0 with a mean of 2.5 (Figure 2). Nine progeny isolates showed average disease reactions greater than FGO (3.3) on Skiff, whereas 32 progeny showed average disease reactions less than SG1 (2.2) (Figure 2). Reaction types of the FGO × SG1 progeny isolates inoculated on Skiff were found to be significantly different than a normal distribution at *P* = 0.05, with more avirulent reactions among the progeny.

Average disease reactions of progeny isolates inoculated on 81-82/033 ranged from 1.2 to 4.0 with a mean of 2.4 (Figure 2). Ten of the progeny showed average disease reactions greater than FGO (3.2) on 81-82/033 and only two of the progeny showed average disease reactions less than SG1 (1.5) (Figure 2). Reaction types of the FGO × SG1 progeny isolates inoculated on 81-82/033 were found to be significantly different than a normal distribution at *P* = 0.05 among the progeny.

Average disease reactions on TR326 ranged from 2.0 to 4.3 with a mean of 3.3 (Figure 2). Twenty-one of the progeny isolates showed average disease reactions greater than FGO (3.7) on TR326, and two isolates exhibited average disease reactions less than SG1 (2.3). Reaction types of the FGO × SG1 progeny isolates inoculated on TR326 were normally distributed (*P* = 0.05) among the progeny.

Average disease reactions on PI 392501 ranged from 1.5 to 4.3 with a mean of 3.3 (Figure 2). Only two of the progeny had an average disease reaction greater than FGO (4.2) when inoculated on PI 392501, and only four isolates exhibited average disease reactions less than SG1 (2.5) (Figure 2). Reaction types of the FGO × SG1 progeny isolates inoculated on PI 392501 were normally distributed (*P* = 0.05) among the progeny.

### Genotyping, SNP identification, and genetic mapping results

Ion Torrent RAD-GBS sequencing for the *P*. *teres* f. *maculata* FGO × SG1 progeny isolates resulted in an average of 178,438 reads per progeny isolate with a minimum and maximum of 57,037 and 763,764 reads, respectively. For parental isolates FGO and SG1, we obtained 444,012 and 439,723 reads, respectively. RAD-GBS sequencing data generated reads with an average read length of 145 bp across the FGO × SG1 progeny isolates.

Initial filtering based on the max quality score threshold of 999 assigned by SAMtools generated 6156 markers. Allele frequency cutoff filtering further reduced the marker data set to 5736. Marker data were lastly filtered for missing data >30%, resulting in 1034 quality SNP markers for generating genetic maps.

Genetic mapping used 983 of the 1034 filtered SNP markers, resulting in 16 linkage groups ranging in size from 13 to 221 cM with a total map size of 1807 cM (Table 2). Using the draft genome sequence of FGO (40.5 Mb) as a size estimate of the *P*. *teres* f. *maculata* genome, the physical to genetic distance ratio in the FGO × SG1 population was ∼24.5 kb/cM. To improve downstream QTL analysis, the lowest quality cosegregating markers were eliminated from the data set leaving 488 independent SNP markers exported to Qgene 4.3.10 (Joehanes and Nelson 2008) for identification of markers associated with *P*. *teres* f. *maculata* virulence/avirulence.

**Table 2:** FGO × SG1 *P*. *teres* f. *maculata* summary of genetic maps

Linkage Group	Markers*^a^*	Linkage Group Size (cM)	Average Marker Density (cM/Marker)
1.1	129 (64)	221.4	3.5
1.2	147 (74)	210.0	2.8
2.1	124 (60)	199.3	3.3
3.1	92 (45)	157.5	3.5
4.1	78 (37)	169.7	4.6
5.1	103 (52)	170.1	3.3
5.2	7 (3)	24.7	8.2
6.1	30 (10)	56.2	5.6
7.1	35 (21)	133.3	6.3
8.1	66 (31)	132.9	4.3
8.2	5 (4)	20.5	5.1
9.1	49 (29)	79.2	2.7
9.2	11 (4)	13.6	3.4
10.1	50 (21)	81.5	3.9
10.2	6 (2)	14.3	7.2
11.1	51 (31)	122.7	4.0
Total	983 (488)	1806.9	—

Summary table of genetic maps of the FGO × SG1 *P*. *teres* f. *maculata* population including linkage groups, number of markers, and average marker density.

a Total number of SNP markers on each linkage group, parentheses show marker numbers after cosegregating markers are removed.

### QTL analysis

**Figure 3:**
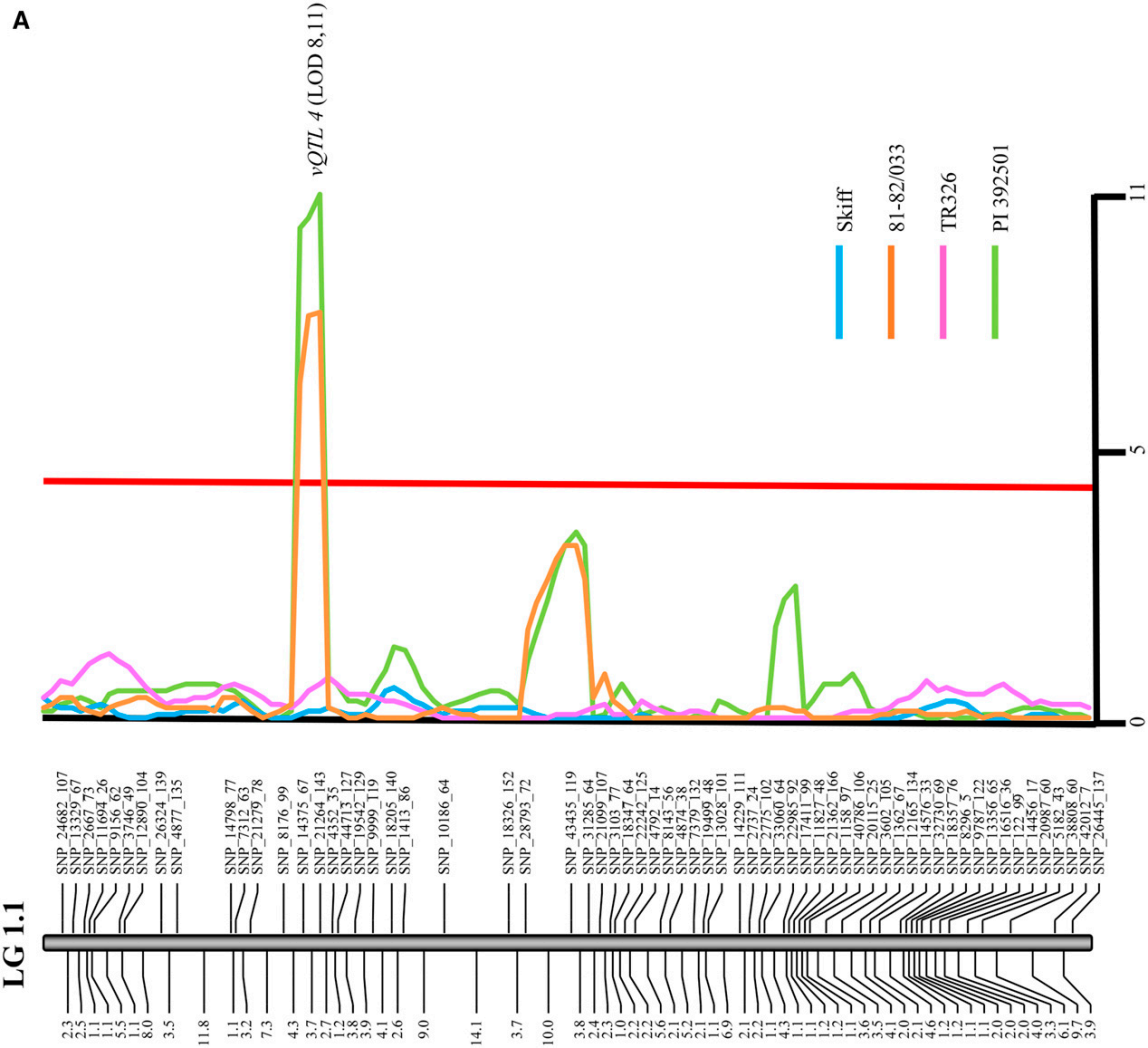

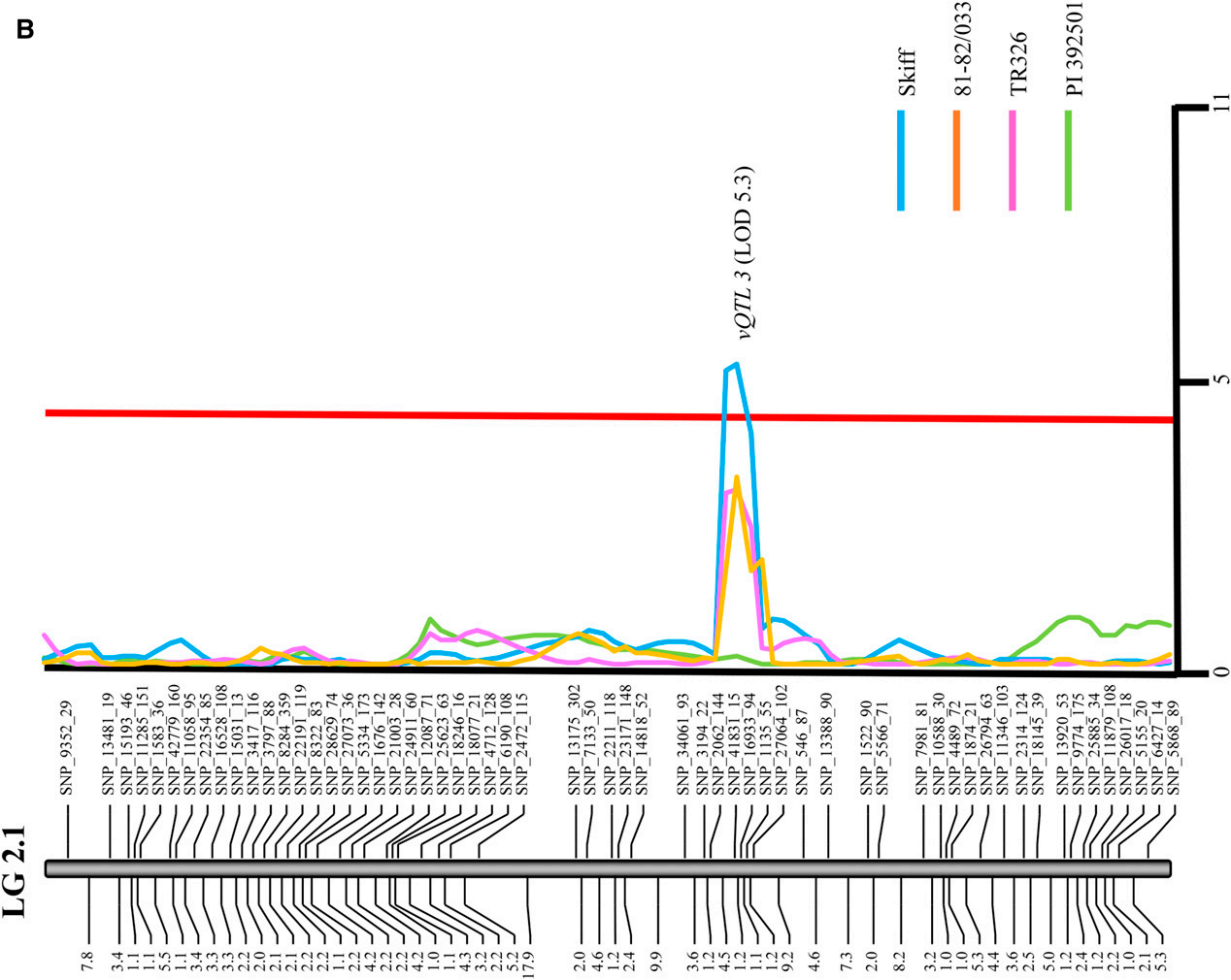

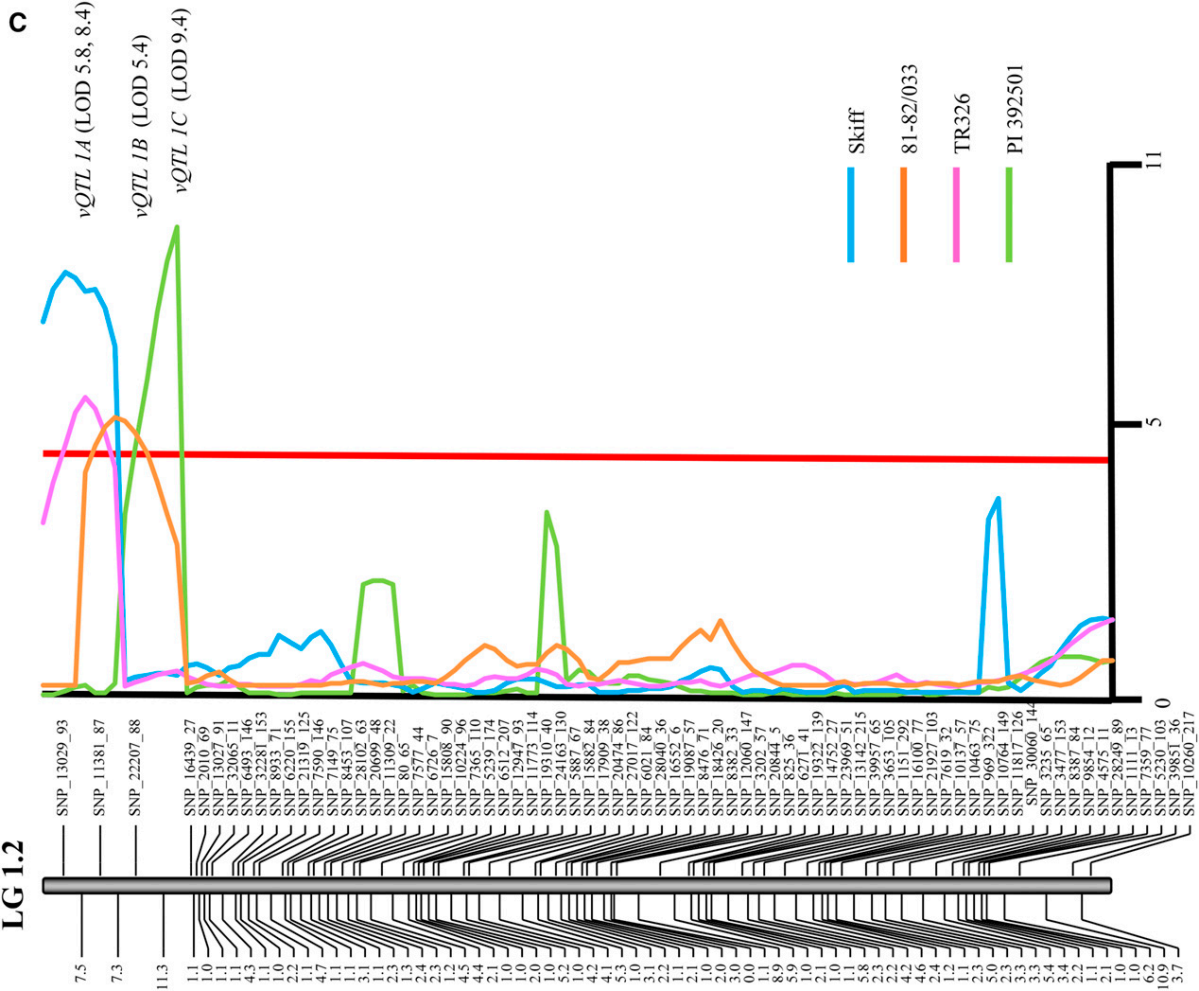

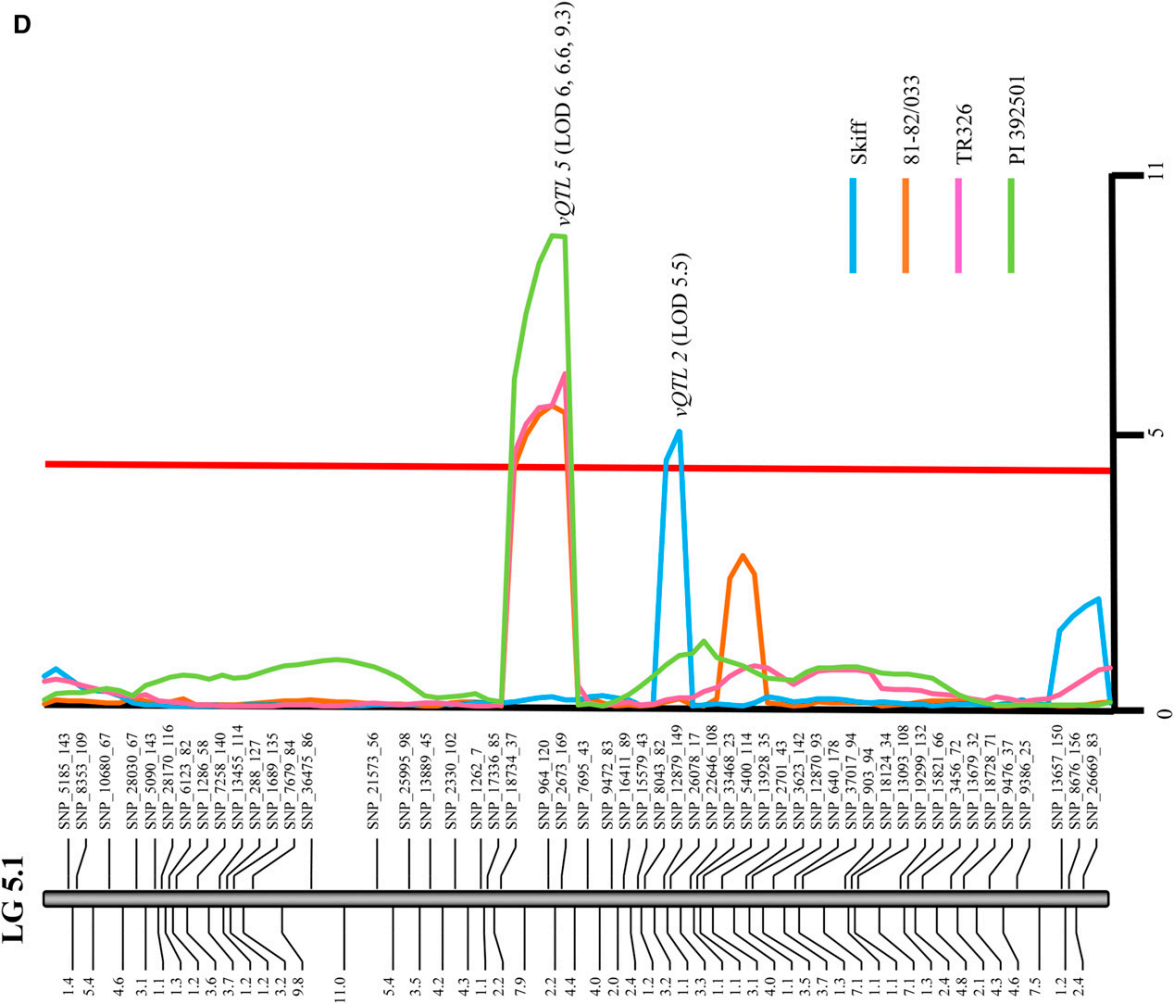

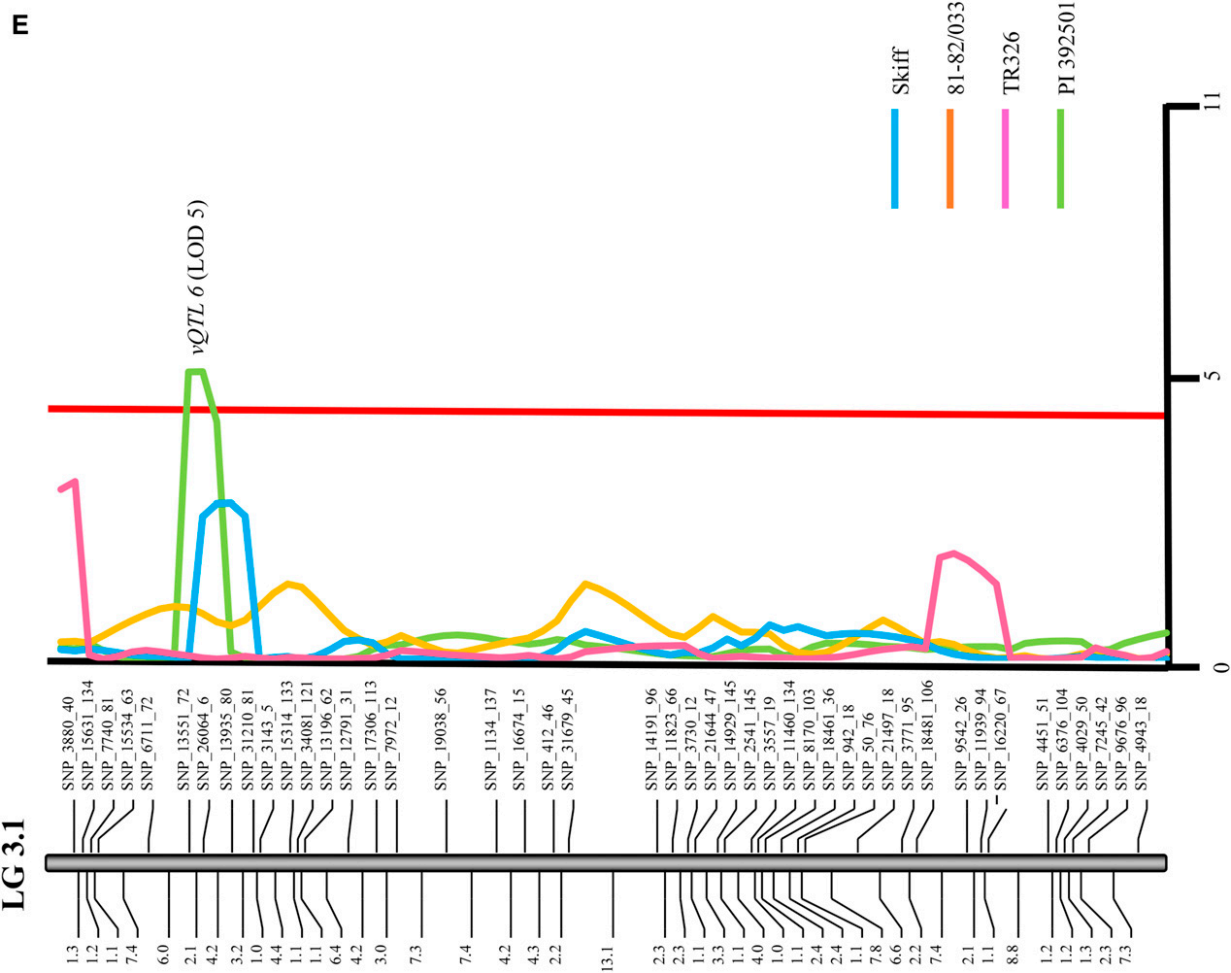
QTL analysis of *P. teres* f. *maculata* virulence in the FGO × SG1 fungal population on four of the differential lines (Skiff, 81/82-033, TR326, and PI392501) for (A) LG 1.1, (B) LG 2.1, (C) LG 1.2, (D) LG 5.1, and (E) LG 3.1. The *x*-axis shows the position of the QTL composite interval mapping regression curve on its respective linkage group. For each linkage group, genetic distances are on the bottom in centimorgans with SNP markers shown above. The *y*-axis shows LOD values with the red line denoting the significant LOD threshold (4.0). QTL associated with individual barley lines ranged from two to four with each line used showing a different QTL pattern. QTL analysis identified six genomic regions showing association to *P. teres* f. *maculata* virulence. LOD, logarithm of the odds; QTL, quantitative trait loci; SNP, single nucleotide polymorphism.

Using a 0.01 *P*-value, LOD permutations yielded a calculated LOD threshold range of 3.6–4.0 for the four data sets and therefore the critical LOD threshold was adjusted to 4.0. When progeny isolates were inoculated on barley genotype Skiff, three QTL associated with virulence were identified with the LOD values above the 4.0 LOD threshold (Figure 3 and Table 3). The most significant QTL (*vQTL 1A*) (Figure 3C) accounted for 31% of the phenotypic variation and was identified on LG 1.2. A second QTL (*vQTL 2*) (Figure 3D) unique to the Skiff data set was identified on LG 5.1 and accounted for 22% of the phenotypic variation. A third QTL (*vQTL 3*) (Figure 3B) identified on LG 2.1 accounted for 20% of the phenotypic variation. All QTL significant on Skiff at the LOD 4.0 threshold were contributed by FGO.

**Table 3:** Identified quantitative trait loci (*vQTL*) in the FGO × SG1 population

*vQTL*	Linkage Group	Lines	LOD	*R*^2^
*vQTL 1A*	LG 1.2	Skiff, TR326	8.4/5.8	0.31/0.23
*vQTL 1B*	LG 1.2	81-82/033	5.4	0.21
*vQTL 1C*	LG 1.2	PI 392501	9.4	0.34
*vQTL 2*	LG 5.1	Skiff	5.5	0.22
*vQTL 3*	LG 2.1	Skiff	5.3	0.20
*vQTL 4**^a^*	LG 1.1	81-82/033, PI 392501	8.0/11.0	0.30/0.37
*vQTL 5*	LG 5.1	81-82/033, TR326, PI 392501	6.0/6.6/9.3	0.33/0.26/0.34
*vQTL 6*	LG 3.1	PI 392501	5.0	0.20

Summary table of *vQTL* identified for the FGO × SG1 pathogen population on barley genotypes: Skiff, 81-82/033, TR326, and PI 392501. QTL is shown in the first column, followed by the linkage group, the lines the QTL was identified on, the respective LOD score, and the *R*^2^ for each QTL identified. vQTL, virulence quantitative trait loci; LOD, logarithm of the odds; LG, linkage group.

a *vQTL 4* was the only virulence contributed by SG1.

Inoculation data collected from barley genotype 81-82/033 resulted in the identification of three QTL above the 4.0 LOD threshold (Table 3). The most significant QTL (*vQTL 4*) (Figure 3A), accounting for 30% of the phenotypic variation, was identified on LG 1.1. A second QTL accounting for 21% of the phenotypic variation was located on LG 1.2. This QTL was in close proximity to *vQTL 1A* from Skiff, with a noticeable overlap between these QTL regions, and was therefore named *vQTL 1B* (Figure 3C). A third QTL (*vQTL 5*) (Figure 3D) identified on LG 5.1 accounted for 33% of the phenotypic variation. Significant QTL were contributed by both parents, with *vQTL 4* contributed by SG1, and *vQTL 1B* and *vQTL 5* being contributed by FGO. The six QTL identified in the evaluation of barley genotypes Skiff and 81-82/033 represent different genomic regions associated with *P*. *teres* f. *maculata* virulence, with only the overlap of *vQTL 1A* and *vQTL 1B* observed between the two barley genotypes.

Inoculation of progeny isolates on TR326 resulted in two major QTL above the 4.0 LOD threshold (Table 3). The most significant QTL (*vQTL 5*) accounted for 26% of the phenotypic variation and was observed on LG 5.1 (Figure 3D). This region is the same *vQTL 5* region that was identified on 81-82/033. The second largest QTL (*vQTL 1A*) was identified on LG 1.2 (Figure 3C) and accounted for 23% of the phenotypic variation. Again, *vQTL 1A* is at the same position as *vQTL 1A* identified on Skiff. TR326 did not show any unique QTL, with all of its QTL being identified in either 81-82/033 or Skiff.

Inoculation data collected on barley genotype PI 392501 resulted in four QTL above the 4.0 LOD threshold (Table 3). The most significant QTL (*vQTL 4*) was identified on LG 1.1 (Figure 3A) and accounted for 37% of the phenotypic variation. *vQTL 4* was also identified with 81-82/033, making the only SG1-contributed virulence QTL common between PI 392501 and 81-82/033. The second QTL (*vQTL 1C*) was identified on LG 1.2 (Figure 3C) and accounted for 34% of the phenotypic variation. *vQTL 1C* is also in close proximity to *vQTL 1A* and *1B* on Skiff and 81-82/033, respectively, with a noticeable overlap within the QTL regions. The third QTL (*vQTL 5*) located on LG 5.1(Figure 3D) accounted for 34% of the phenotypic variation. *vQTL 5* was common among 81-82/033, TR326, and PI 392501 with varying degrees of significance, with the greatest phenotypic variation resulting with PI 392501 (34%) (Figure 3). The fourth QTL (*vQTL 6*), which was unique to PI 392501, was located on LG 3.1 (Figure 3E) and accounted for 20% of the phenotypic variation.

## Discussion

To our knowledge, this is the first report genetically characterizing the virulence of the SFNB pathogen *P. teres* f. *maculata*. Virulence and avirulence have been defined in different ways, and in this study virulence was defined as the amount of disease caused and is measured by a quantitative 1–5 disease rating scale. In this study, we did not observe any strong differential responses that have been reported in the *P. teres* f. *teres* interaction, but rather saw quantitative contribution of several virulence loci that contributed incrementally to the disease phenotype, resulting in bell-shaped curves (Figure 2) rather than having bimodal distribution as would be expected for a qualitative trait.

Skiff has been used in SFNB differential studies and has been proposed for the international SFNB differential set (McLean *et al.* 2014). Skiff was resistant to isolates collected in Australia and the US (Karki and Sharp 1986; Tekauz 1990; Arabi *et al.* 1992; McLean *et al.* 2010a,b, 2012; Gupta *et al.* 2011); however, the Skiff resistance has been overcome in both these geographical regions (McLean *et al.* 2012). Skiff is moderately resistant to the Australian isolate SG1 (average disease reaction type = 2.2) but susceptible to the North Dakota isolate FGO (average disease reaction type = 3.3), but the average disease reaction types across the population ranged from 1.5 to 4.0 showing 41 progeny isolates with transgressive segregation, 32 lower than 2.2, and 9 higher than 3.3. Because all QTL significant at the LOD 4.0 threshold (*P* = 0.01) were contributed by the FGO parent, we looked at virulence QTL between LOD 3.1 (*i.e.*, *P* = 0.05) and 4.0 (*P* = 0.01) for relatively minor effect virulences that were contributed by the SG1 parent. A relatively minor effect QTL (LOD 3.8) with the virulence allele contributed by SG1 was identified (data not shown), providing a possible explanation for the transgressive segregation in this interaction.

Barley genotype 81-82/033 has also been used in differential studies (McLean *et al.* 2012), harbored the highest level of resistance to SG1 (1.5), and was moderately susceptible to FGO (3.2). Transgressive segregation was also present for this line, especially on the virulent end of the population with average disease reactions reaching as high as 4.0. This transgressive segregation among the progeny can easily be explained by the fact that at the QTL with the highest effect (*vQTL* 4), the virulence allele is conferred by SG1, whereas with the other two QTL the virulence allele is contributed by FGO. In addition to the three major effect QTL, two other QTL identified at the 0.05 *P*-value cutoff showed virulences that were contributed by FGO (data not shown), resulting in a total of five virulence loci, showing the highly quantitative nature of this interaction.

Barley genotype TR326 has also been proposed as a barley line in the international SFNB differential set (McLean *et al.* 2014) with resistance to some Australian isolates, including moderate resistance to SG1 (average disease reaction = 2.3), but was susceptible to isolate FGO with an average disease reaction of 3.7. The average disease reactions ranged from 2.0 to 4.3, with very few progeny outside of the parental reaction types. Using a 0.05 *P*-value cutoff, an additional QTL was identified (data not shown), with the virulence allele being contributed by FGO. Two of the progeny isolates have a higher average disease reaction than the virulent parent FGO. Therefore, it is likely that one or more undetected virulences contributed by SG1 are responsible for the transgressive segregation.

Barley genotype PI 392501 was chosen based on an evaluation of a barley core collection with both FGO and SG1 (Neupane *et al.* 2015). PI 392501 showed an average disease reaction of 2.5 on SG1 and 4.2 on FGO, with average disease reactions across progeny ranging from 1.5 to 4.3. QTL analysis of the PI 392501 data set showed a similar QTL pattern as 81-82/033, with QTL showing up in similar locations on LG 1.1, 1.2, and 5.1; however, a unique QTL with the virulence allele contributed by FGO was present on LG 3.1.

A total of eight QTL were identified, with three of these being present in a closely linked region on LG 1.2. It is likely that a single gene underlies all three of the LG1.2 QTL, but it is also possible that this region harbors several genes that are contributing to virulence and are each specific to their corresponding barley genotype. Therefore, we labeled these three QTL as *vQTL 1A*, *B*, and *C*. *vQTL 3* on LG 5.1 is specific to three of the four barley genotypes indicating that PI 392501, TR326, and 81-82/033 all likely harbor a similar susceptibility gene specific to an FGO virulence factor; however, Skiff does not. Similarly, *vQTL 4* on LG 1.1 is common between PI 392501 and 81-82/033, but not TR326 or Skiff. Interestingly, this virulence is conferred by the less virulent parent SG1, providing a reason for the transgressive segregation of virulence in the population.

The results presented here indicate several things about the SFNB interaction. For each of the barley lines inoculated, increasing the number of virulence alleles at the vQTL in a progeny isolate incrementally increases the average disease reaction score across the population. This in itself provides strong evidence that, on these four barley lines, the *P. teres* f. *maculata*–barley interaction is not a gene-for-gene interaction involving pathogen avirulence factors and dominant host resistance. Closely related cereal pathogens including *P. tritici-repentis* (Ciuffetti *et al.* 2010), *Parastagonospora nodorum* (Friesen *et al.* 2008; Liu *et al.* 2012), and *P. teres* f. *teres* (Liu *et al.* 2015) at least partially follow a NE-triggered susceptibility model. In these interactions, it has been shown that the pathogen secretes NEs that interact directly or indirectly with host targets to induce necrosis, a result of programmed cell death (PCD). Unlike the biotrophic gene-for-gene interaction, where recognition results in a defense response leading to resistance, the pathogens mentioned above are known to induce “recognition” resulting in the same hallmarks of the defense response, including PCD, but with the outcome being a compatible or susceptible reaction where the pathogen can penetrate, feed, and sporulate. Additional research is necessary, including the functional characterization of the genes underlying these *vQTL* before conclusions can be drawn on how *P. teres* f. *maculata* is interacting with its host.

Given an average physical to genetic distance of 24.5 kb/cM, this mapping population will be useful in the map-based cloning of the virulence genes underlying each QTL reported. When looking at markers flanking the QTL, the candidate gene regions range from 250 to 450 kb (data not shown), providing manageable genomic regions. Additionally, several effector-like genes have been identified and prioritized as candidates within these QTL. Validation of these genes will give us the additional tools needed for a more in-depth understanding of this host–pathogen interaction. This work will also lead to a better understanding of the recent virulence shifts that have made this pathogen an economically significant problem in several barley producing regions of the world.

The data presented here represent the first *P. teres* f. *maculata* mapping population, first genetic maps of *P. teres* f. *maculata*, and the first genetic characterization of pathogen virulence. It is with this research that we provide the first look at characterizing and therefore understanding the complex structure of pathogen virulence and how this virulence is interacting with known sources of resistance/susceptibility. Given the observed complexity underlying the FGO × SG1 biparental population, it is almost certain that the complexity present in the natural population is significantly greater. With the unknown level of virulence complexity present in the natural population, there is much more work to be done to characterize and understand this disease system.

## Supplementary Material

Supplemental material is available online at www.g3journal.org/lookup/suppl/doi:10.1534/g3.117.043265/-/DC1.
